# The Specific Effects of OD-1, a Peptide Activator, on Voltage-Gated Sodium Current and Seizure Susceptibility

**DOI:** 10.3390/ijms21218254

**Published:** 2020-11-04

**Authors:** Ming-Chi Lai, Sheng-Nan Wu, Chin-Wei Huang

**Affiliations:** 1 Department of Pediatrics, Chi-Mei Medical Center, Tainan 710, Taiwan; vickylai621@gmail.com; 2 Department of Physiology, College of Medicine, National Cheng Kung University, Tainan 701, Taiwan; 3 Department of Neurology, National Cheng Kung University Hospital, College of Medicine, National Cheng Kung University, Tainan 701, Taiwan

**Keywords:** OD-1, scorpion toxin, voltage-gated Na^+^ current, action current, seizure, neuronal excitability

## Abstract

OD-1, a scorpion toxin, has been previously recognized as an activator of voltage-gated Na^+^ currents. To what extent this agent can alter hippocampal neuronal Na^+^ currents and network excitability and how it can be applied to neuronal hyperexcitability research remains unclear. With the aid of patch-clamp technology, it was revealed that, in mHippoE-14 hippocampal neurons, OD-1 produced a concentration-, time-, and state-dependent rise in the peak amplitude of *I*_Na_. It shifted the *I*_Na_ inactivation curve to a less negative potential and increased the frequency of spontaneous action currents. Further characterization of neuronal excitability revealed higher excitability in the hippocampal slices treated with OD-1 as compared with the control slices. A stereotaxic intrahippocampal injection of OD-1 generated a significantly higher frequency of spontaneous seizures and epileptiform discharges compared with intraperitoneal injection of lithium-pilocarpine- or kainic acid-induced epilepsy, with comparable pathological changes. Carbamazepine significantly attenuated OD-1 induced seizures and epileptiform discharges. The OD-1-mediated modifications of *I*_Na_ altered the electrical activity of neurons in vivo and OD-1 could potentially serve as a novel seizure and excitotoxicity model.

## 1. Introduction

OD-1, a scorpion toxin, was originally isolated from the venom of the Iranian yellow scorpion (*Odonthobuthus doriae*) and was characterized as an α-like toxin [1]. It was previously reported to be a strong activator of rat Na_V_1.7, human Na_V_1.4, and rat Na_V_1.6 channels, and Na_V_1.5/β_1_ Na_V_1.7/β1 or Na_V_1.8/β1 currents expressed in *Xenopus* oocytes [2,3,4,5]. Despite its ability to induce pain (e.g., OD-1 envenoming), whether OD-1 can interact with endogenous Na_V_ channels to modify the amplitude or gating of voltage-gated Na^+^ currents (*I*_Na_) in native electrically excitable cells remains unclear [3,6,7].

Voltage-gated Na^+^ (Na_V_) channels are essential for the generation and propagation of action potentials (APs) in electrically excitable membranes. There are nine Na_V_ channel α-subunits (Na_V_1.1–1.9) expressed among excitable mammalian tissues, including the central and peripheral nervous systems, the endocrine system, skeletal muscle, and the heart [8,9,10,11,12,13]. The Na_V_ channel protein contains four homologous domains (D1–D4), each consisting of a six transmembrane domain (S1–S6) and a reentry P loop between S5 and S6. Upon brief depolarization, Na_V_ channels go through a rapid transition from their resting state to the open state and then to the inactivated state. Genetic defects that cause Na_V_ channel inactivation and result in sustained Na^+^ currents after AP firing have been recognized as having severe consequences, such as seizures and epilepsy, periodic paralysis, paramyotonia, and LQT-3 syndrome [14,15,16,17].

Traditional animal models of seizure and epilepsy lack good specificity to receptors and proteins that are involved in wide-spectrum brain pathologies and excitotoxicity, which leads to substantial difficulties in evaluating specific ion channels or receptors. This is especially true for chemical or drug-induced chronic epilepsy models, such as pilocarpine-induced epilepsy and acute seizure models, such as PTZ-induced seizures. Some previous studies have suggested that the prominent role of systemic inflammation in epilepsy could be closely related to the use of these traditional models [18,19], which further raises concern about fever-related seizures [20]. Furthermore, high mortality rates when seizures are induced and low success rates for the production of animals with spontaneous recurrent seizures using these traditional models exemplify the need for a new model.

Previous studies have shown that low nanomolar concentrations of the OD-1 toxin impair the steady-state fast inactivation process, enhance recovery from fast inactivation, increase the peak Na^+^ current, and give rise to a substantial persistent Na^+^ current [2], which supports the hypothesis that OD-1 activation could be suitable for the development of a novel epilepsy and seizure model.

The current study is intended to explore the possible effects of OD-1 on ionic currents, particularly on endogenous *I*_Na_ in mHippoE-14 hippocampal neurons. This cell line is known to have the characteristics of hippocampal neurons and enables accurate in-vitro assays for use in the discovery, development, and validation of new therapeutics targeted to central nervous system disorders. Since hippocampal neuronal aberrant excitability in human intractable temporal lobe epilepsy has been well established [21], and abnormal epileptiform discharges have been well described in the human hippocampus based on patients with temporal lobe epilepsy [22], the structural and cellular mechanisms that cause the hippocampus to be chronically hyperexcitable have thus been heavily linked to epileptogenesis [23]. Furthermore, the potential role of OD-1 as a seizure-inducing agent has also been compared with the traditional pilocarpine and kainic acid (KA)-induced seizure models. The results provide evidence suggesting that the presence of OD-1 stimulates *I*_Na_ in a concentration- and time-dependent manner in mHippoE-14 hippocampal cells and hippocampal CA1 pyramidal neurons. OD-1-mediated activation of *I*_Na_ is therefore expected to contribute to an increase in spontaneous AP frequency, and thus its accentuation of excitotoxicity could serve as a novel seizure model.

## 2. Results

### 2.1. Effect of OD-1 on the Voltage-Gated Na^+^ Current (I_Na_)

**Figure 1:**
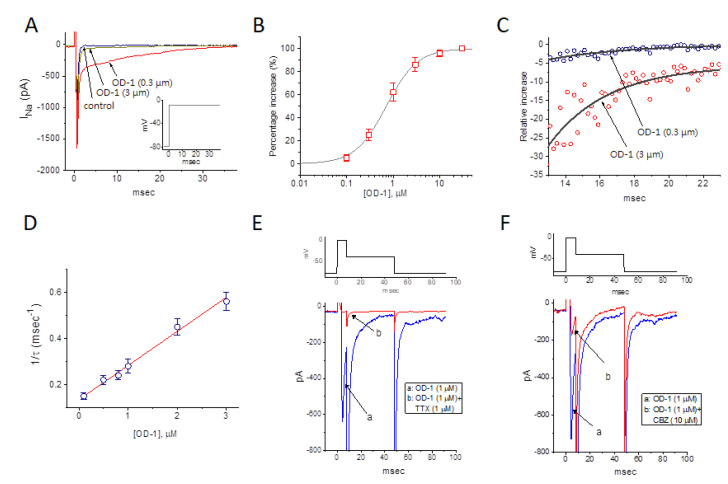
Stimulatory effect of OD-1 on voltage-gated Na^+^ current (*I*_Na_). In these experiments, cells were bathed in Ca^2+^-free Tyrode’s solution, and the recording pipette was filled with a Cs^+^-containing solution. (**A**) Representative *I*_Na_ traces were obtained in the control (control) and during exposure to 0.3 μM OD-1 and 3 μM OD-1. The inset indicates the voltage protocol used. (**B**) Concentration-dependent effect of OD-1 on the peak amplitude of *I*_Na_ (mean ± SEM; *n* = 9–11 for each point). The peak amplitude of *I*_Na_ in response to brief membrane depolarization from −80 to −10 mV was measured. The peak *I*_Na_ during exposure to 30 μM OD-1 was taken to be 100%. The continuous line represents the best fit of the data points to the sigmoidal Hill equation, as detailed in the Materials and Methods section. The EC_50_ values and Hill coefficient were 0.71 μM and 1.3, respectively. (**C**) Time courses of the relative increase in *I*_Na_ following treatment with 0.3 μM OD-1 (a) and 3 μM OD-1 (b). The trajectory in the presence of 0.3 μM OD-1 and 3 μM OD-1 was fitted using a single exponential (as indicated by smooth curves) with a value of 4.62 and 3.43 msec, respectively. The relative increase (i.e., (*I*_control_-*I*_OD-1_)/*I*_control_) was evaluated by dividing the OD-1-sensitive current by the current obtained in the control. (**D**) The relationship of the reciprocal to the time constant (i.e., 1/τ) of the relative increase versus the OD-1 concentration was plotted (mean ± SEM; *n* = 9–12 for each point). The data points shown in the open circles were fitted using a linear regression and indicated that there was a molecularity of one. On the basis of the binding scheme, the forward (*k*_+1_*) and backward (*k*_−1_) rate constants for OD-1-induced stimulation of *I*_Na_ were calculated to be 0.148 msec^−1^μM^−1^ and 0.135 msec^−1^, respectively. (**E**) Effect of OD-1 and OD-1 plus tetrodotoxin (TTX) on voltage-gated Na^+^ current in mHippoE-14 neurons. Cells were bathed in Ca^2+^-free, Tyrode’s solution, and the pipette was filled with a Cs^+^-containing solution. The current trace labeled “a” was obtained in the presence of 1 μM OD-1, while that labeled “b” was obtained after further addition of 1 μM TTX, but still in the presence of 1 μM OD-1. The upper part shows the applied voltage-clamp protocol. (**F**) Effect of OD-1 and OD-1 plus carbamazepine (CBZ) on voltage-gated Na^+^ current in mHippoE-14 neurons. Cells were bathed in Ca^2+^-free, Tyrode’s solution, and the pipette was filled with a Cs^+^-containing solution. The current trace labeled “a” was obtained in the presence of 1 μM OD-1, while that labeled “b” was obtained after further addition of 10 μM CBZ, but still in the presence of 1 μM OD-1. The upper part shows the voltage-clamp protocol applied. Data were analyzed using a one-way ANOVA with the least-significant difference post hoc comparisons.

In the first set of experiments, the recording pipette was filled with a Cs^+^-containing solution, and the effect of OD-1 on *I*_Na_ was determined in cells immersed in a Ca^2+^-free Tyrode’s solution. The composition of the solution is described in the Materials and Methods section of this work. Under whole-cell current recordings, the cell being examined was maintained at −80 mV, and a brief depolarizing pulse to −10 was applied to evoke *I*_Na_, the biophysical properties of which has been described previously [24]. As the cells were exposed to OD-1, the peak amplitude of *I*_Na_ was progressively increased, and the concomitant inactivation time course of the current slowed (Figure 1). As cells were exposed to 3 μM OD-1, the peak *I*_Na_ amplitude in response to rapid depolarization significantly increased from 1.19 ± 0.32 to 1.66 ± 0.67 nA (*n* = 11, *p* < 0.05). In addition, the slow component of the inactivation time constants of *I*_Na_ in response to brief membrane depolarization was significantly prolonged to 45.6 ± 4.7 msec (*n* = 11, *p* < 0.05) from a control of 8.1 ± 1.1 msec (*n* = 11). After washout of OD-1, the current amplitude returned to 1.28 ± 0.39 pA (*n* = 9, *p* < 0.05).

As the OD-1 concentration was increased, it caused a progressive increase in the peak *I*_Na_ amplitude elicited in response to rapid membrane depolarization (Figure 1A,B). The association between OD-1 concentration and the percentage increase in peak amplitude were then determined. Notably, OD-1 was able to effectively raise the peak amplitude of *I*_Na_ in a concentration-dependent manner (Figure 1B). A least-squares fit to the Hill function, as detailed in the Materials and Methods, yielded 0.71 μM as the concentration required for half-maximal stimulation (i.e., EC_50_) and a Hill coefficient of 1.3.

### 2.2. Kinetic Study of OD-1-Induced Stimulation of I_Na_

To provide a quantitative estimate of the OD-1-induced increase in *I*_Na_, the time constants for the relative increase in *I*_Na_ (i.e., (*I*_control_-*I*_OD-1_)/*I*_control_) observed in the cells were further analyzed. The time courses for the relative increase in the presence of different OD-1 concentrations were fitted to a single-exponential function (Figure 1C). The concentration dependence of the *I*_Na_ decay elicited by rapid membrane depolarization is illustrated in Figure 1D. It is clear from these results that the addition of OD-1 produced a concentration-dependent increase in the rate (1/τ) of the relative increase in *I*_Na_. For example, as cells were depolarized from −80 to −10 mV, the time constant (τ) of the *I*_Na_ relative increase obtained in the presence of 1 and 3 μM OD-1 were fitted to a single exponential with values of 3.57 ± 0.11 msec (*n* = 9) and 1.79 ± 0.09 msec (*n* = 10), respectively.

It should be noted that OD-1 resulted in a concentration-dependent rise in the inactivation rate of *I*_Na_ in response to brief membrane depolarization. It can thus be hypothesized that the stimulatory effect of OD-1 on *I*_Na_ can be explained as a state-dependent stimulator that acts on the open state of the channel on the basis of a minimal kinetic scheme, as described in the Materials and Methods section. As such, forward and backward rate constants, *k*_+1_*·(OD-1) and *k*_−1_, were determined from the τ values obtained for the different OD-1 concentrations (Figure 1D). The resultant forward or backward rate constants were estimated to be 0.148 msec^−1^ μM^−1^ and 0.135 msec^−1^, respectively. From these rate constants, a dissociation constant (*K*_D_) value of 0.91 μM was determined to be required for OD-1-induced stimulation of *I*_Na_. This value was close to the EC_50_ value described above. Furthermore, the value of free energy (i.e., G = −RTln(*K*_eq_), where *K*_eq_ (equilibrium constant) = 1/*K*_D_) required for the OD-1-mediated stimulation of *I*_Na_ was calculated to be 34.0 kJ/mol.

Tetrodotoxin (1 μM) completely blocked the OD-1-mediated effect on *I_Na_*. In the presence of OD-1 plus TTX, the peak amplitude of *I*_Na_ was decreased to 29 ± 8 pA (*n* = 7, *p* < 0.05) from a control value (i.e., in the presence of 1 μM OD-1 for 634 ± 28 pA, *n* = 7; Figure 1E). Furthermore, in the presence of OD-1 plus CBZ, the peak amplitude of *I*_Na_ was decreased to 164 ± 19 pA (*n* = 7, *p* < 0.05) from a control value (i.e., in the presence of 1 μM OD-1) for 728 ± 34 pA (*n* = 7; Figure 1F).

### 2.3. Stimulatory Effect of OD-1 on the Conductance–Voltage Relationship of Peak I_Na_

**Figure 2:**
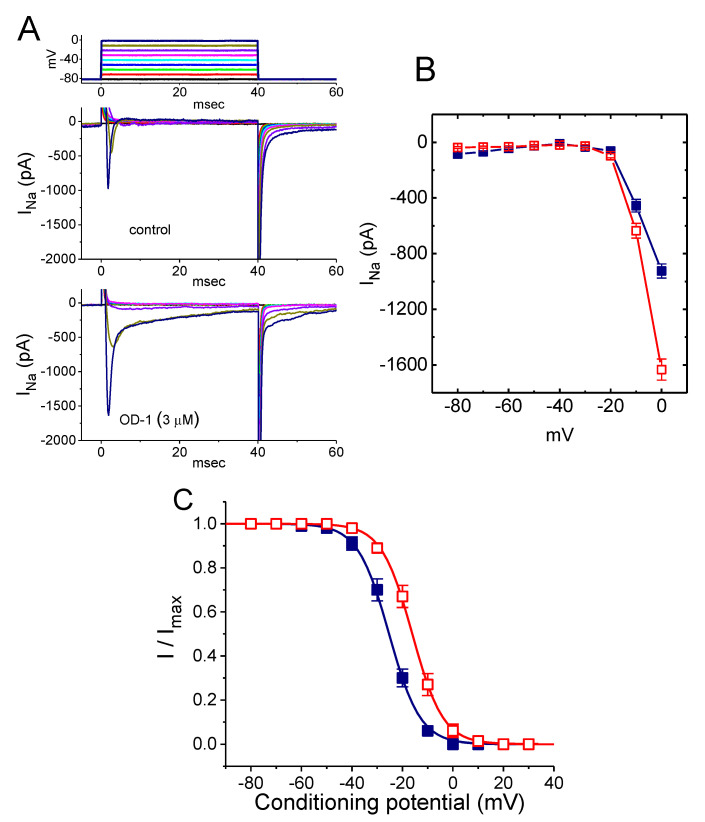
Average conductance–voltage relationship (**A**,**B**) and steady-state inactivation curves (**C**) of peak *I*_Na_ obtained with or without the addition of OD-1. The experiments were conducted in cells bathed with Ca^2+^-free Tyrode’s solution, and the recording pipette was filled with a Cs^+^-containing solution. (**A**) Representative *I*_Na_ traces evoked by different voltage steps are shown in the uppermost part. The upper part indicates current traces obtained in the control, while the lower part is those taken during exposure to 3 μM OD-1. (**B**) Average conductance–voltage relationship of peak *I*_Na_ in the absence (■) and presence (□) of 3 μM OD-1 (mean ± SEM; *n* = 9–11 for each point). Notably, the presence of OD-1 increased the peak conductance of *I*_Na_ with no change in the overall conductance-versus-voltage relationship to the current. (**C**) Steady-state inactivation curve of *I*_Na_ obtained with and without the addition of 3 μM OD-1 (mean ± SEM; *n* = 9 for each point; absence (■) and presence (□) of 3 μM OD-1). Data were analyzed using a one-way ANOVA with the least-significant difference post hoc comparisons.

To characterize the stimulatory effect of OD-1 on peak *I*_Na_, we attempted to determine whether it might modify the conductance–voltage relationship of *I*_Na_. Figure 2A,B illustrates the average relationship of conductance versus voltage achieved in the absence and presence of OD-1 (3 μM). Cell exposure to OD-1 (3 μM) significantly increased the whole cell conductance of peak *I*_Na_ measured at voltages ranging between −10 and 20 mV. However, the overall conductance–voltage relationship of peak *I*_Na_ was not modified in the presence of OD-1.

### 2.4. Effect of OD-1 on the Steady-State Inactivation Curve of I_Na_

The steady-state inactivation curve of *I*_Na_ with or without the addition of OD-1 was further characterized. In these experiments, cells were bathed in Ca^2+^-free Tyrode’s solution, and the steady-state inactivation parameters of *I*_Na_ were then quantitatively obtained in the absence or presence of 3 μM OD-1. As shown in Figure 2C, the normalized amplitude of peak *I*_Na_ was plotted against the conditioning potential, and the continuous lines were well fitted by the Boltzmann equation, as described in the Materials and Methods section. The values of *V*_1/2_ and *q* were −25.3 ± 1.1 mV and 6.2 ± 0.9 *e* (*n* = 9), respectively, for the control data at −16.1 ± 1.2 mV and 6.1 ± 0.9 *e* (*n* = 9) following the addition of OD-1 (3 μM). The results revealed that the presence of OD-1 caused a significant shift in the inactivation curve along the voltage axis towards a more depolarized potential by approximately 9 mV; however, there was no modification in the gating charge of the curve.

### 2.5. Effects of OD-1 on Spontaneous ACs

**Figure 3:**
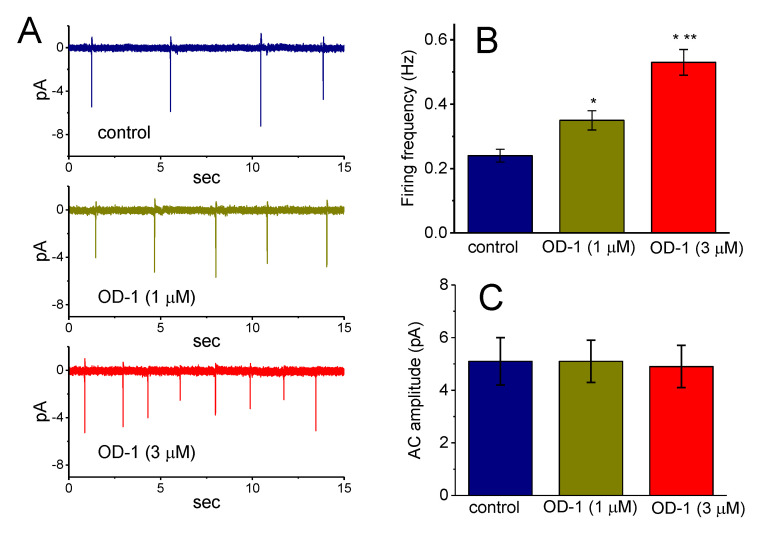
Stimulatory effect of OD-1 on AC frequency and amplitude. Cell-attached voltage-clamp current recordings were made in these experiments. The recording pipette was filled with a K^+^-containing solution, and once the cell-attached model was clearly achieved, the potential was maintained at the resting potential of the cell (−70 mV). (**A**) Representative current traces obtained in the absence (upper) and presence of 1 μM OD-1 (middle) and 3 μM OD-1 (lower). The results regarding the addition of OD-1 were obtained 2 min after the cells were exposed to the compound (1 or 3 μM). Note that the trace displaying inward deflections reflects the emergence of ACs. Summary bar graphs showing the effect of OD-1 on the frequency and amplitude of spontaneous ACs are shown in (**B**) and (**C**), respectively, (mean ± SEM; *n* = 11). * Significantly different from the control group (*p* < 0.05). ** Significantly different from OD-1 (1 μM; *p* < 0.05). Data were analyzed using a one-way ANOVA with the least-significant difference post hoc comparisons.

In another set of experiments, we attempted to explore the effect of OD-1 on spontaneous ACs (action currents). The cells were bathed in normal Tyrode’s solution containing 1.8 mM CaCl_2_. Consistent with previous observations [25], it was possible to detect the occurrence of spontaneous ACs since cell-attached voltage-clamp current recordings were firmly established in these cells. The downward deflection appearing in the current traces shown in Figure 3A indicates the capacitive current that charges the surface membrane. Notably, when OD-1 was applied to the bath, a progressive increase in the frequency of spontaneous ACs could be clearly observed. Figure 3B,C are summary bar graphs showing the stimulatory effect of OD-1 (1 or 3 μM) on the firing frequency and amplitude of the ACs, respectively. It is therefore possible that the OD-1-induced rise in the alternating current frequency is predominantly mediated by its stimulatory action on *I*_Na_; however, no change in the AC amplitude was demonstrated in the presence of OD-1.

### 2.6. OD-1 Enhanced Neuronal Excitability in Hippocampal Slices

**Figure 4:**
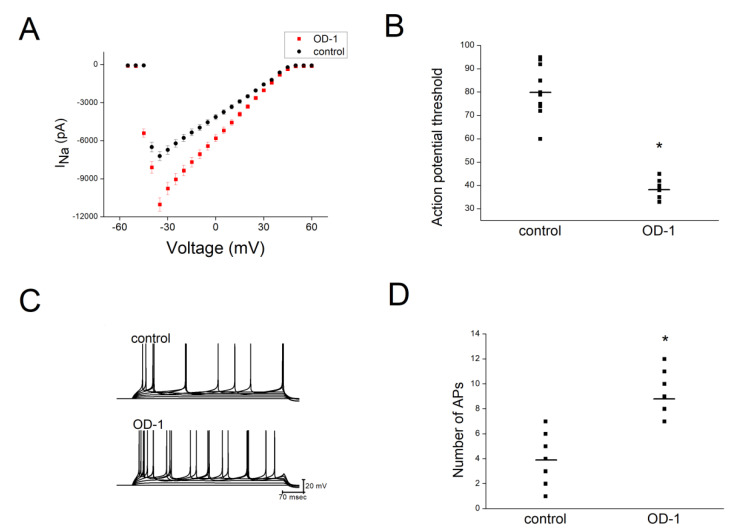
Comparison of hippocampal CA1 neuronal excitability in Sprague Dawley (SD) rats with OD-1 treatment (OD-1 group) and without OD-1 treatment (control group). The OD-1 concentration was 1 μM. (**A**) The *I*_Na_ was significantly higher in the brain slices in the OD-1 group, as compared with the control group. (**B**) The threshold current required one to elicit an AP in the presence and absence of OD-1. (**C**) Representative traces of AP firing in the presence and absence of OD-1. Depolarizing current injection from 0 to 140 pA in 20 pA increments with a duration of 500 msec. Current injection yielded a significantly higher number of APs in the OD-1 group, as compared to the control group. The horizontal lines indicate mean values. (**D**) The number of APs elicited by threshold current injection in the control and with OD-1. The horizontal lines indicate mean values. * Significantly different from the control group (*p* < 0.05). Data were analyzed using an ANOVA Kruskal–Wallis H test, followed by Dunn’s multiple comparison tests (*n* = 7 in each group).

Further analysis of the effect of OD-1 on neuronal excitability was done by comparing CA1 pyramidal neuronal excitability with OD-1 treatment (OD-1 group) and without OD-1 treatment (control) in hippocampal slices. There was a significant increase in the *I*_Na_ in the OD-1 group (1 μM), as compared with the control (Figure 4A). The lowest current amplitude that led to the firing of APs from resting potential was significantly lower in the OD-1 group (38.5 ± 1.21 pA) compared with the control (80.6 ± 3.85 pA; Figure 4B). A current injection yielded a significantly higher number of APs in the OD-1 group (9.1 ± 0.7), as compared with the control group (3.9 ± 0.5; Figure 4C,D).

### 2.7. SRS and Epileptiform Discharges Following a Stereotactic Hippocampal Injection of OD-1

**Figure 5:**
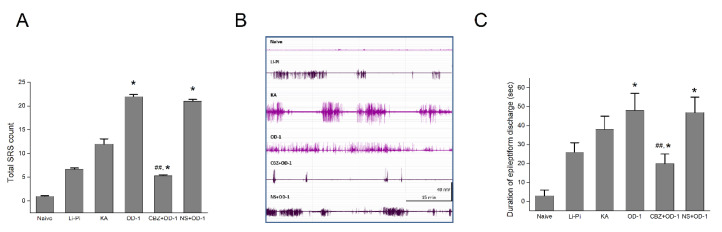
The spontaneous recurrent seizure (SRS) and epileptiform discharges on EEG during the chronic stage following status epilepticus. (**A**) The OD-1 group had a significantly higher total SRS count compared with the naïve, Li-Pi, KA (all * *p* < 0.05), and OD-1 + CBZ groups (^##^ *p* < 0.01). The total SRS count in the KA group was higher than that in the OD-1 + CBZ group (*p* < 0.05). The control group (NS + OD-1) also had a significantly higher total SRS count than the CBZ + OD-1 group. (**B**) and (**C**) Representative figures of the epileptiform discharges on EEG showing that the high frequency epileptiform bursts in rats with SRS were more frequently seen in the OD-1 group and NS + OD-1 group compared with the naïve, Li-Pi, and KA groups (all * *p* < 0.05) and CBZ + OD-1 group (^##^ *p* < 0.01). Similarly, the epileptiform discharges in the KA group was more frequent than that of the CBZ + OD-1 group (*p* < 0.05; * *p* < 0.05, ^##^ *p* < 0.01, *n* = 7 in each group). Data were analyzed using ANOVA followed by Fisher’s least significant difference tests.

The spontaneous recurrent seizures (SRSs) during the chronic stage following status epilepticus were evaluated in the three experimental groups. We also evaluated behavioral seizures and the electroencephalography (EEG) data from a naïve control group that did not undergo lithium-pilocarpine, KA, or OD-1, a subgroup of OD-1 with pretreatment with CBZ, and an additional subgroup of the OD-1 control group with pretreatment with normal saline (NS + OD-1). Behavioral seizures were not observed in the naïve control group. The SRSs observed were at least stage 3 (anterior limb clonus), stage 4 (dorsal extension (rearing)), and stage 5 (loss of balance and falling; Racine scale). The mean duration of the SRS was 1–2 s. With the exception of the total SRS count, we did not find other differences in the seizure presentation or duration among these models. The mortality during acute seizures was 2/9 (22.2%) in all groups. The behavioral seizures were similar, but the OD-1 group had a significantly higher total SRS count, compared with the naïve, Li-Pi, KA, and OD-1 + CBZ groups (naïve: 1.0 ± 0.08, Li-Pi: 6.7 ± 0.24; KA 12.0 ± 1.08; OD-1: 22.0 ± 0.41, OD-1 + CBZ: 5.3 ± 0.24, *n* = 7, all *p* < 0.05; Figure 5A). NS + OD-1 group showed a higher total SRS count similar to the OD-1 group (20 ± 0.47) as compared with the other groups (naïve, Li-Pi, KA, and CBZ + OD-1 group (all *p* < 0.05). The epileptiform discharges on the EEGs were recorded and compared among the groups (naïve, Li-Pi, KA, OD-1, CBZ + OD-1, and NS + OD-1). A representative figure showed that the high frequency epileptiform bursts in the rats with SRS were seen significantly more frequently in the OD-1 group, as compared with the other experimental groups (the duration of epileptiform discharges in naïve: 3 ± 3, Li-Pi: 26 ± 5 s, KA: 38 ± 7 s, the OD-1 group: 48 ± 9 s, the OD-1 + CBZ: 20 ± 5, *p* = 0.012, *n* = 7; Figure 5C). The NS + OD-1 group showed a similar longer duration of epileptiform discharges (47 ± 8 s) compared to the other groups (naïve, Li-Pi, KA, and CBZ + OD-1 group (all *p* < 0.05). It should be noted that both the total SRS count and duration of epileptiform discharge in the OD-1 group were especially significantly higher than those in the OD-1 + CBZ group (*p* < 0.01).

### 2.8. Hippocampal Neuronal Damage and Aberrant Network Following Stereotactic Hippocampal Injection of OD-1

**Figure 6:**
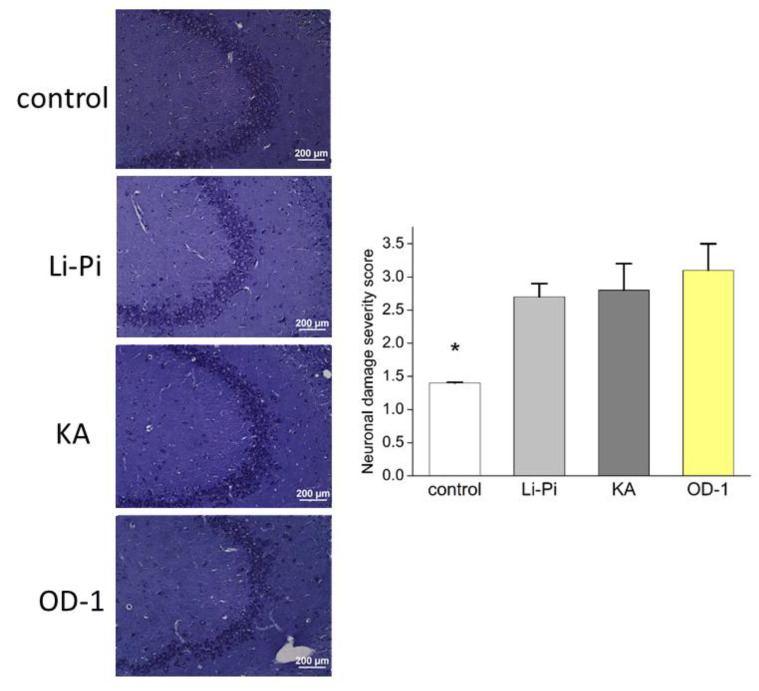
There were similar amounts of cresyl violet stained hippocampal neurons in the Li-Pi, KA, and OD-1 models. Scale bar = 200 μm. The semiquantitative neuronal damage scores were similar across the three groups (*p* = 0.20) although the OD-1 group had a slightly higher overall score. The naïve control (1.4 ± 0.01) showed significantly less damage than the other three groups (* *p* < 0.05). Data were analyzed using an ANOVA Kruskal–Wallis H test followed by Dunn’s multiple comparison tests (*n* = 7 in each group).

**Figure 7:**
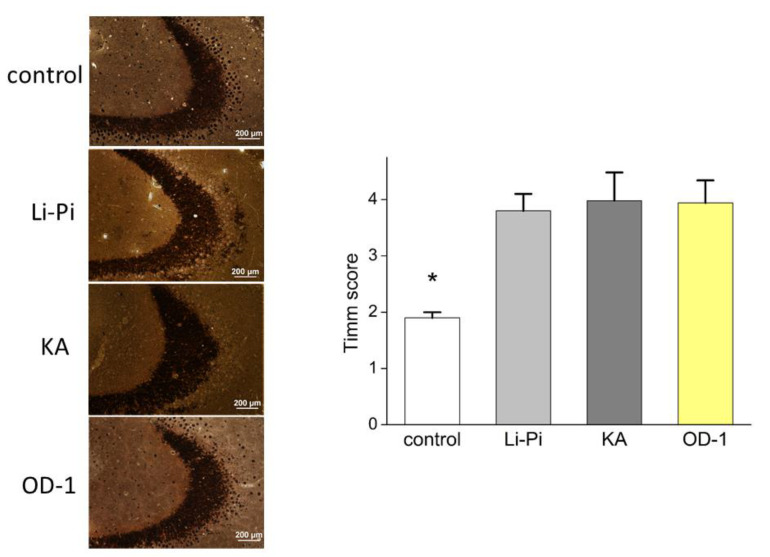
There were similar amounts of cresyl violet stained neurons in the Li-Pi, KA, and OD-1 models. Scale bar = 200 μm. The semiquantitative neuronal damage scores were similar across the three groups (*p* = 0.20) although the OD-1 group had a slightly higher overall score. The naïve control (1.9 ± 0.1) showed significantly less damage than the other three groups (* *p* < 0.05). Data were analyzed using an ANOVA Kruskal–Wallis H test followed by Dunn’s multiple comparison tests (*n* = 7 in each group).

Similar hippocampal neuronal damage was observed across the Li-Pi, KA, and OD-1 models, and they all demonstrated significantly more damage than the naïve control group (Figure 6). The semiquantitative neuronal damage scores were similar in the Nissl staining in the Li-Pi, KA, and OD-1 groups (Li-Pi: 2.7 ± 0.2, KA: 2.8 ± 0.4, and OD-1: 3.1 ± 0.4, *p* = 0.20, Figure 6). However, the OD-1 group had a higher overall score. The naïve control (1.4 ± 0.01) showed significantly less damage than the other three groups (*p* < 0.05). Timm’s staining was used to evaluate the mossy fiber sprouting. The results demonstrated that OD-1 induced similar mossy fiber sprouting to that in Li-Pi and KA, which are the standard epilepsy models (Li-Pi: 3.8 ± 0.3, KA: 3.98 ± 0.5, and OD-1: 3.94 ± 0.4, *p* = 0.67; Figure 7). Similarly, the naïve control (1.9 ± 0.1) showed significantly less damage as compared to the three groups (*p* < 0.05).

## 3. Discussion

The findings of the current study highlighted the effects of OD-1 on the activation of *I*_Na_ in hippocampal neurons and alterations in hippocampal neuronal network excitability. OD-1 was effective in stimulating *I*_Na_ in a concentration- and time-dependent manner from the extracellular surface of the Na_V_ channel. The addition of OD-1 also produced *I*_Na_ activation by shifting the steady-state inactivation curve to a less negative voltage with no distinct change in the gating charge. Stimulation of *I*_Na_ followed by changes in membrane polarization in the presence of OD-1 will affect the firing rate and hormonal and neurotransmitter secretions [11,24,26,27].

OD-1 is a water-soluble peptide with a molecular weight of 7206.1 g/mol, which means it does not rapidly enter the cell interior. Therefore, different from the stimulatory actions of tefluthrin on *I*_Na_ [24,25,28], its effects on Na_V_ channels and spontaneous ACs should be located at the extracellular surface of the channel, although the detailed mechanisms of OD-1 actions on the channel have yet to be fully determined. It is tempting to speculate that the OD-1 molecule either directly modulates the pore-forming α-subunit of the channel or interacts with a closely associated structure via an extracellular site. It should also be noted that, unlike the action of tefluthrin or telmisartan on other types of K^+^ currents that have been reported previously [25,28,29], the amplitude of the delayed-rectifier M-type and *erg*-mediated K^+^ currents were not modified during cell exposure to OD-1 .

In the current study, stimulation of *I*_Na_ by OD-1 tended not to be instantaneous, but rather developed over time after the channels were bound with the compound, thereby producing a conceivable concentration-dependent and exponential increase over the time course of current inactivation (Figure 1C,D). Since the cells were exposed to relatively low concentrations of the agent, the values of EC_50_ or K_D_ required for OD-1-mediated activation of *I*_Na_ appeared to be higher than those required for the suppression of rat Na_V_1.7 or Na_V_1.6 channels [2,5]. However, the transient (i.e., peak *I*_Na_) and late *I*_Na_ suggest that OD-1 at a low concentration may depress the repolarization phase of neuron and endocrine or neuroendocrine cells following an AP. The experimental results of cell-attached voltage-clamp recordings revealed that the presence of OD-1 raised the frequency of spontaneous ACs.

Previous studies have demonstrated OD-1′s ability to alter the amplitude and gating of Na_V_ currents [2,3,4,6,7]. The Na_V_1.2, Na_V_1.3, Na_V_1.6-α, and Na_V_1.7-α subunits were expressed in hippocampal neurons [30,31,32,33]. It is therefore conceivable that OD-1 could interact with Na_V_ channels to modify the amplitude and gating of *I*_Na_ in hippocampal neurons. Supportively, the antiseizure effects of some of the Na_V_1.7 inhibitors, lidocaine and carbamazepine, have been well demonstrated, both experimentally and clinically [34]. Our animal experiments with OD-1 + CBZ further support the Na_V_1.7 agonism of OD-1, demonstrating that OD-1 causes epileptic seizures predominantly through its effect on the Na_V_1.7. Importantly, this peptide and other structurally similar peptides (e.g., AaH II, BmK M1, Lqh-2, or Lqh-3) would therefore be expected to be powerful tools through which new ligands for extensive investigations into Na_V_ channels, such as structure–activity studies of the Na_V_ channel, may be generated.

There were some potential confounding factors on OD-1 associations with altered neuronal excitability. Different anesthetics may influence the propagation of action potentials. In our experimental setting, both the OD-1 and control groups were from the same animals having undergone the same anesthesia. Therefore, the differential effect of anesthetics would not be a major concern in this study. In addition, the solvent (polyoxyethylene sorbitan monooleate) we used for CBZ did not induce overt effects that would influence the animal’s behavior, as the CBZ + OD-1 group showed a marked reduction in seizure count and epileptiform discharges on EEG compared to the OD-1 group.

Our in vivo studies on seizure modeling showed that OD-1 was comparable to traditional seizure models, which use Li-Pi and KA, in terms of seizure, epileptiform discharges on EEGs, and pathological neuronal damage. Although the frequency of SRS and epileptiform discharges were higher in the OD-1 group, similar pathological changes, including neuronal loss and mossy fiber sprouting, supported the notion that focal intrahippocampal stereotaxic injections of OD-1 could induce focal hippocampal damage that is comparable with a traditional chronic epilepsy model created by intraperitoneal injection of pilocarpine or KA and undergoing status epilepticus. The avoidance of a systemic response, including active inflammation and widespread neurotoxic effects, could be beneficial in the selection of seizure modeling. In addition, it has been reported that intracranial injections of KA could potentially lead to more focal injuries and a low proportion of animals generating SRS [35]. Furthermore, the higher frequency of recurrent seizures and epileptiform discharges in the OD-1 group could be useful as a potential model for epilepsy and seizure research, including use as a potential screening model for specific sodium-modulating antiepileptic drugs.

## 4. Materials and Methods

All experiments, including the animal procedures, were reviewed and approved by the Institutional Animal Care and Use Committee (IACUC) (Approval No: 107128, Date: 5 February 2018) at National Cheng Kung University, Tainan, Taiwan. All efforts were made to reduce the total number of rats used.

### 4.1. Chemicals and Solutions

OD-1 (C_308_H_466_N_90_O_95_S_8_, GVRDAYIADDKNCVYTCAS-NGYCNTECTKNGAESGYCQWIGRYGNACWCIKLPDEVPIRIPG-KCR) was obtained from Tocris Cookson Ltd. (Bristol, UK); carbamazepine and tefluthrin were obtained from Sigma-Aldrich (St. Louis, MO, USA). Unless stated otherwise, the tissue culture media, horse serum, fetal bovine and calf serums, L-glutamine, and trypsin/EDTA were obtained from Invitrogen (Carlsbad, CA, USA). The stock solution of OD-1 was vigorously vortexed to ensure complete solubilization before the experiments were performed. All other chemicals, such as CsCl, CsOH, aspartic acid, and HEPES, were of the best available quality, most of which were of analytical grade.

The composition of the HEPES-buffered normal Tyrode’s solution was as follows (in mM): NaCl 136.5, KCl 5.4, CaCl_2_ 1.8, MgCl_2_ 0.53, glucose 5.5, and HEPES-NaOH buffer 5.5 (pH 7.4). To record action currents (ACs) or K^+^ currents, the recording pipettes were filled with the following solution (in mM): K-aspartate 130, KCl 20, KH_2_PO_4_ 1, MgCl_2_ 1, EGTA 0.1, Na_2_ATP 3, Na_2_GTP 0.1, and HEPES-KOH buffer 5 (pH 7.2). To measure the Na^+^ currents, K^+^ ions inside the pipette solution were replaced with equimolar Cs^+^ ions, and the pH value of the solution was adjusted to 7.2 with CsOH. The pipette solution was filtered on the day of use with a 0.22-μm pore size syringe filter (Millipore, Burlington, MA, USA).

### 4.2. Cell Preparations

The embryonic mouse hippocampal cell line (mHippoE-14, CLU198) was obtained from Cedarlane CELLutions Biosystems, Inc. (Burlington, ON, Canada) [36,37,38]. Cells were maintained in Dulbecco’s modified Eagle’s medium supplemented with 10% fetal bovine serum (*v*/*v*) and 2 mM L-glutamine. The experiments were performed 5 or 6 days after the cells were cultured to 60–80% confluence.

### 4.3. Electrophysiological Measurements

As in our previous experiments, mHippoE-14 neurons were harvested and transferred to a recording chamber positioned on the stage of an inverted microscope, which was coupled to a digital video camera (DCR-TRV30; Sony, Tokyo, Japan). The cells were immersed at room temperature (20–25 °C) in normal Tyrode’s solution. Patch clamp recordings under the whole-cell mode were achieved with either an RK-400 (Bio-Logic, Claix, France) or an Axopatch 200B amplifier (Molecular Devices, Sunnyvale, CA, USA) [39]. Patch electrodes with a tip resistance of 3–5 MΩ were pulled from Kimax-51 glass capillaries (#34500; Kimble, Vineland, NJ, USA) on either a PP-830 vertical puller (Narishige, Tokyo, Japan) or a P-97 horizontal puller (Sutter, Novato, CA). The signals, including voltage and current tracings, were stored online at 10 kHz in an ASUSPRO-BU401LG computer (ASUS, Taipei City, Taiwan) that was controlled using pClamp 10.7 software (Molecular Devices), as described previously [39,40].

Spontaneous ACs that represented the occurrence of APs were measured by means of cell-attached voltage-clamp recordings, as described previously [24]. AC measurements were made to allow quantification of the underlying AP frequency under conditions where the intracellular milieu was left relatively unchanged [24,25,41]. The capacitive current, which can be measured when the cell fires an AP, appeared as a brief spike in the downward direction.

### 4.4. Data Analyses

To evaluate the effect of concentration-dependent stimulation of OD-1 on the peak amplitude of *I*_Na_, the mHippoE-14 neurons were immersed in Ca^2+^-free Tyrode’s solution. Each examined cell was clamped at −80 mV, and a 40-msec depolarizing pulse to −10 mV was delivered. The peak amplitude of *I*_Na_ measured during cell exposure to 30 μM OD-1 was taken as 100%, and the current amplitudes were then compared to those after the addition of 10 μM OD-1. The concentration required to increase the current amplitude by 50% was determined by the use of the Hill function:

(1) $$\mathrm{Percentage}\;\mathrm{increase}\;\left(\%\right)=\frac{E_{\max}+{\left[C\right]}^{n_{H}}}{{\mathrm{EC}}_{50}^{n_{H}}+{\left[C\right]}^{n_{H}}},$$

where [C] is the OD-1 concentration; E_max_ is the maximal increase in the peak *I*_Na_ caused by OD-1; EC_50_ is the concentration required for 50% stimulation; and n_H_ is the Hill coefficient.

The stimulatory effect of OD-1 on *I*_Na_ is explained using a state-dependent blocker that preferentially binds to the Na_V_ channel in its open state. A minimal kinetic scheme was derived as follows:

(2) $$\frac{dC}{dt}=O*\beta -C*\alpha \;\frac{dO}{dt}=C*\alpha +O\cdot OD1*k_{-1}-O*\beta -O*k_{+1}^{*}\cdot \left[OD1\right]\;\frac{d\left(O\cdot OD1\right)}{dt}=O*k_{+1}^{*}\cdot \left[OD1\right]-O\cdot OD1*k_{-1}$$

where [OD-1] is the OD-1 concentration, and α and β are the voltage-gated rate constants for the opening and closing of the Na_V_ channels, respectively. *k*_+1_* and *k*_−1_ are the forward and backward rate constants of OD-1, respectively, while C, O, and O·OD-1 are the closed, open, and open-activated states, respectively.

The forward (i.e., on or *k*_+1_*) and backward (i.e., off or *k*_−1_) rate constants were determined from the time constant (τ) of the relative increase (i.e., (*I*_control_-*I*_OD-1_)/*I*_control_) in *I*_Na_ obtained using different OD-1 concentrations [41,42]. According to the minimal binding scheme, these rate constants could then be estimated using the following equation:

(3) $$\frac{1}{\tau }=k_{+1}^{*}\times \left[C\right]+k_{-1}$$

where *k*_+1_* and *k*_−1_ are respectively derived from the slope and from the *y*-axis intercept at [C] = 0 of the linear regression interpolating the reciprocal time constants (1/τ) versus the OD-1 concentration, and [C] is the OD-1 concentration used.

The steady-state inactivation curves of *I*_Na_ without and with the addition of OD-1 were constructed and plotted against the conditioning potential and then fit to the Boltzmann equation as follows:

(4) $$I=\frac{I_{max}}{1+e^{\frac{-\left(V-V_{1/2}\right)qF}{RT}}},$$

where *I*_max_ is the maximal amplitude of *I*_Na_ in the absence or presence of OD-1 (3 μM); *V* is the conditioning potential in mV; *V*_1/2_ is the membrane potential at which half-maximal inactivation of *I*_Na_ is achieved; *q* is the apparent inactivation gating charge; *F* is Faraday’s constant; *R* is the universal gas constant; and *T* is the absolute temperature.

### 4.5. Animal Experiments

Adult Sprague–Dawley male rats weighing 180–200 g were purchased from National Cheng Kung University. They were housed in the university’s Animal Center and allowed free access to water and a pelleted rodent diet (Richmond Standard; PMI Feeds, St Louis, MO, USA). All efforts were made to reduce the number of rats used.

### 4.6. Preparation of Brain Slices and Patch Clamp Technology

Hippocampal slices were prepared from the rats, which were anesthetized using urethane and then decapitated. The brains were removed and placed in ice-cold artificial cerebrospinal fluid (ACSF) containing 126 mM NaCl, 2.5 mM KCl, 2.0 mM MgCl_2_, 2.0 mM CaCl_2_, 1.25 mM NaH_2_PO_4_, 26 mM NaHCO_3_, and 10 mM D-glucose. Transverse hemisectional slices (350 μm-thick) of the hippocampus were then obtained, and the slices were incubated at room temperature for >1 h before being transferred to the recording chamber with fresh ACSF (containing the same components as described previously). All solutions were saturated with 95% O_2_/5% CO_2_.

Whole cell patch clamp recordings were performed in the CA1 pyramidal neurons using a MultiClamp 700B amplifier. Patch electrodes (3–5 MΩ) were pulled from 1.5 mm outer diameter thin-walled glass capillaries in three stages and were filled with intracellular solutions containing 123 mM K-gluconate, 17 mM KCl, 10 mM HEPES, 1.1 mM EGTA, 0.1 mM CaCl_2_, and 2 mM Na_2_-ATP (pH 7.25, osmolarity 290–300). Input resistance was measured before and after each recording, and any recording with >25% change in input resistance was discarded. Signals were acquired via a Digidata 1440A analog-to-digital interface; they were low-pass filtered at 2 kHz and digitized at 10 kHz [42].

*I*_Na_ from the neurons were recorded using the voltage-clamp mode with a holding potential of −60 mV; they were elicited using a 200 ms step depolarization ranging from −60 to +60 mV in 5 mV increments. The neuronal firing pattern was recorded based on the membrane potentials under the current-clamp mode and evoked with 600 ms injection currents ranging from −150 to +140 pA. The intensity of the applied current stimulus was based on the input resistance of the neurons. Since 130 pA was the current threshold for the neurons, the firing number of APs was calculated from those elicited by the +130 pA injection current. The rheobase was measured as the lowest current amplitude that led to firing of APs from the resting potential.

### 4.7. Animal Seizure Modeling

The rats were mainly divided into three experimental groups (OD-1, lithium-pilocarpine (Li-Pi), and KA) and a naive (control) group, which did not undergo Li-Pi, KA, or OD-1 treatments. We also added a subgroup of OD-1 with a specific Nav1.7 antagonist, carbamazepine (CBZ) pretreatment (50 mg/kg, ip) 30 min before the intrahippocampal injection of OD-1 (CBZ + OD-1), and an additional control group with normal saline (ip) 30 min before injection of OD-1 (NS + OD-1). The Li-Pi group was injected with lithium chloride (3 meq/kg; intraperitoneal (ip)), and methylscopolamine (25 mg/kg; subcutaneous (sc)) before they were subjected to pilocarpine (60 mg/kg; sc)-induced seizures, while the KA group was injected with kainic acid (18 mg/kg; ip). The OD-1 group was stereotactically injected with 5 ng OD-1 (12 mouse LD50) in the hippocampus (coordinates 4.1 mm caudal, 3.9 mm lateral to the bregma, and 3.8 mm below the cortical surface) under general anesthesia. KA, lithium, and pilocarpine were dissolved in 0.9% saline. CBZ was prepared as a suspension in 0.5% Tween 80 (polyoxyethylene sorbitan monooleate). In the Li-Pi and KA groups, the behavioral characteristics of the rats during acute epileptic seizures were similar to those reported elsewhere [42,43]. The rats were given zoletil (50 mg/kg, ip), xylazine (20 mg, ip), and atropine (0.2 mg/kg, sc) to diminish the seizures if their epilepticus status lasted for 20 min [43]. Mortality was calculated during the first 24 h after seizure onset. All rats were continuously monitored for the first 24 h after they achieved epilepticus status by two experienced research assistants. The rats were given supportive care: body temperature maintenance with a resistive heating system, food, and adequate hydration with normal saline (0.9% *w/v* of NaCl, 308 mOsm/L). Any animals showing intense signs of acute respiratory distress were immediately euthanized by overdosing with sodium pentobarbital (150 mg/kg, ip).

### 4.8. Spontaneous Recurrent Seizure (SRS)

Monitoring for spontaneous recurring seizures was performed using video cameras mounted above the cages. This was started 7 days after status epilepticus in the Li-Pi and KA groups, and 7 days after intrahippocampal injection of OD-1 in the OD-1 group. It was carried out for 8 h per day over 5 consecutive days [44]. A trained technician, blinded to the experimental design, examined the videos for seizures (i.e., running, jumping, rearing, lordosis, and erect tail). When seizure-like activity was observed, the video was reviewed to confirm seizures.

### 4.9. Electroencephalography (EEG)

The rats were secured in a stereotactic head frame (KOPF Model 900 Small Animal Stereotaxic Instrument) under chlorhydrate (400 mg/kg, ip) anesthesia. Using an aseptic technique, a midline incision was made over the cranium and small burr holes were drilled bilaterally at the cortex for electrode placement (telemetry units (DSI model TL10M3-F50-EEE magnet-activated transmitters; Transoma Medical)). The ground electrode was a fully bared screw placed over the midline cerebellum. EEG recordings were done using the IX-100B Data Acquisition System (iWorx) sampled between 0 and 30 Hz for 2 h. Seizures were identified by characteristic sharp and rhythmic patterns consisting of repeated epileptiform discharges, followed by suppression of background activities. All epileptiform discharges were recorded and analyzed.

### 4.10. Histopathology

#### 4.10.1. Cresyl Violet Staining

Cresyl violet staining was performed to evaluate neuronal loss in animals that had undergone acute seizures and status epilepticus. On day 14, some of the rats’ brains were removed and stored at −80 °C. In NaCl 0.9%, and then paraformaldehyde was used for perfusion. Coronal sections (20 μm thick) of the hippocampus were fixed in formaldehyde, as previously described [45,46]. The cresyl violet-stained sections (10 μm thick) were examined for gross indications of damage to the hippocampus. The severity of neuron loss in different subfields of the hippocampus was scored semiquantitatively as follows: 0 = no neuron loss; 1 ≤ 10% neuron loss, 2 = between 11% and 50% neuron loss, and 3 = a ≥ 51% neuron loss [35,46]. Counts were made at 400× magnification using the Image Plus 2.0 computer image analysis system (Motic, Richmond, BC, Canada). The hippocampal subfields were defined by an imaginary line connecting the tips of the granule cell layer blades, which separated the Cornu Ammonis3c (CA3c; medially) from the CA3b (laterally) and the CA2 from the CA1 [42,45]. Values from the different groups were determined by an investigator who was blinded to the study design, after which the values were averaged for each group.

#### 4.10.2. Timm’s Staining

To compare the mossy fiber sprouting in each group, Na_2_S followed by paraformaldehyde was used for perfusion because of the consideration of Timm staining. On day 14, the remainder of the rats’ brains was removed and coronal sections (20 μm thick) through the entire hippocampus were cut on a Leica CM1900 freezing microtome. Every sixth section was stained with Timm’s stain [42,46]. The sections were developed in the dark for 10–45 min in a 200-mL solution containing 5.1 g citric acid, 4.7 g sodium citrate, 3.47 g hydroquinone, 212.25 mg AgNO_3_, and 120 mL of 50% gum arabic. Timm’s staining was assessed from the septal area to the temporal hippocampus (the region between 2.8 and 3.8 mm posterior to the bregma). A semiquantitative scale was used to evaluate the degree of mossy fiber sprouting in the pyramidal and infrapyramidal areas of the hippocampus CA3 region, and in the granular cell and inner molecular layers of the dentate gyrus [45,46]. The score criteria were 0: no granules, 1: occasional discrete granule bundles, 2: occasional-to-moderate granules, 3: prominent granules, 4: a prominent nearly continuous granule band, and 5: a continuous or nearly continuous dense granule band.

#### 4.10.3. Statistical Analyses

The linear or nonlinear curve fitting to the data sets was appropriately achieved with a least-squares minimization procedure using either the Microsoft Solver function embedded in Excel (Microsoft) or OriginPro 2016 programs (OriginLab). The averaged results are presented as the mean ± standard error of the mean (SEM) with sample sizes (*n*) indicating the cell number from which the experimental results were achieved. A paired or unpaired Student’s *t*-test and a one-way analysis of variance (ANOVA) with the least-significant difference post hoc for multiple comparisons or the Kruskal–Wallis H test followed by Dunn’s multiple comparison tests were utilized for the statistical evaluations. Statistical analyses were performed using the SPSS 20 statistical software package (IBM Corp., Armonk, NY, USA). Statistical significance was demonstrated at a *p*-value of <0.05.

## 5. Conclusions

The present in vitro and in vivo study demonstrated that OD-1-mediated modifications of the amplitude and gating of *I*_Na_ can be expected to alter electrical behaviors in neurons in vivo and that OD-1 may potentially serve as a novel seizure model.

## Abbreviations

AC	Action current
AP	Action potential
EC_50_	50% stimulatory concentration
*I–V*	current versus voltage
*I*_Na_	voltage-gated Na^+^ current
*K*_D_	dissociation constant
Na_V_	voltage-gated Na^+^ channel
SEM	standard error of mean
τ	time constant
